# Drug resistance mutations of hepatitis B virus (HBV) influence the yield of the virions of the human hepatitis delta virus (HDV) and their infectivity

**DOI:** 10.1128/jvi.00610-26

**Published:** 2026-07-13

**Authors:** Oleksandra Chazova, Igor Zaiets, Severin O. Gudima

**Affiliations:** 1 Department of Microbiology, Molecular Genetics and Immunology, University of Kansas Medical Center 21638, Kansas City, Kansas, USA; Wake Forest University School of Medicine, Winston-Salem, North Carolina, USA

**Keywords:** HBV drug resistance mutations, drug-selected mutations in HBsAg, anti-HBV treatments affecting HDV life cycle, HDV assembly and infectivity

## Abstract

This work analyzed 30 drug resistance mutations (DRMs) in the surface antigen of hepatitis B virus (HBV) (i.e., HBsAg) selected by several FDA-approved anti-HBV nucleos(t)ide analogs. All DRM-bearing HBsAg variants supported the formation and secretion of hepatitis delta virus (HDV), and only 13/30 DRMs considerably decreased the yield of secreted HDV virions. However, the majority of DRMs (23/30) greatly reduced HDV infectivity, and four of them even caused the complete loss of infectivity, which was determined by analyzing the levels of HDV RNA genomes in the infected cells, and the fraction of HDV-infected cells. The ability to inhibit HDV infectivity was observed for DRMs located in the large cytosolic loop (LCL), major antigenic loop (MAL), HDV-binding site (HDV-BS), and the transmembrane domains II, III, and IV of HBsAg, which suggested that DRMs mediate their inhibitory influence by both direct and indirect mechanisms that could alter the attachment/entry, post-entry trafficking, and/or subsequent uncoating of the virions. This report also established the transmembrane domains II, III, and IV as critical regulators of the overall infectivity of HDV virions. It became apparent that in the settings of natural HBV/HDV infection, DRMs can therefore greatly reduce the spread/superinfection of HDV and diminish the size of the HDV reservoir in chronically infected livers. Overall, our findings advanced the understanding of how anti-HBV drug treatments influence the HDV life cycle, the roles of HBV envelope proteins in facilitating the assembly and infectivity of HDV, and the mechanism of complex HBV-HDV interactions.

**IMPORTANCE:**

The study showed that the drug resistance mutations (DRMs) in the surface antigen of hepatitis B virus (HBsAg) can greatly reduce hepatitis delta virus (HDV) assembly and infectivity. Since most of the tested DRMs considerably reduced HDV infectivity, and four of them resulted in a complete loss of the infectivity, such effects can lead to suppression of HDV spread/superinfection and reduction of the HDV reservoir in the infected liver. If most detrimental for infectivity DRMs are present on the majority of HBsAg molecules, this could facilitate substantial suppression of chronic HDV infection and possibly reduce HDV-associated liver pathogenesis. It was also determined that DRMs located in the transmembrane domains II, III, and IV of HBsAg could critically alter HDV infectivity. Our findings therefore suggest that different HBsAg regions work in the intramolecular coordinating concert, facilitating the assembly and infectivity of the virions, and that certain short- and long-distance interactions between different regions of HBsAg take place.

## INTRODUCTION

**Fig 1:**
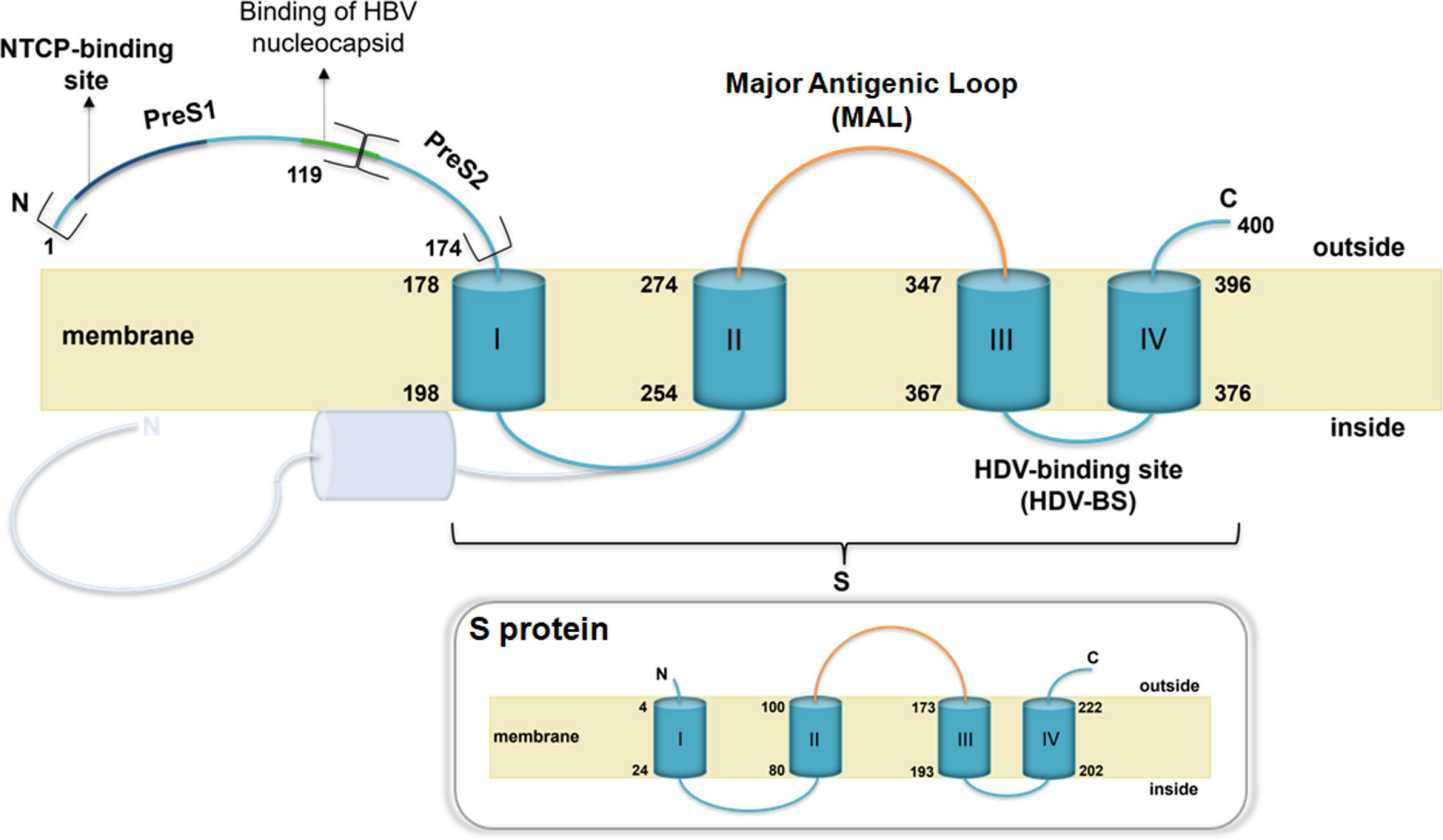
Structure and topology of the large envelope protein of HBV. On top is the large (L) envelope protein of HBV that is shown embedded in the virion’s membrane. The membrane and the spaces inside and outside the virion are indicated. The N- and C-termini of the L protein are marked. Two topologies of the L protein are depicted. The boundaries of the different domains of the L protein are shown. For the infection-competent topology, the important structural elements on the outside of the virion are the PreS1 and PreS2 domains, and the MAL. In this case, the L protein can interact with the HBV receptor NTCP located on the outer surface of a susceptible hepatocyte. The other domains are the TMDI, TMDII, TMDIII, and TMDIV, and two cytosolic loops inside the virion, the LCL between TMDI and TMDII, and the small cytosolic loop between TMDIII and TMDIV, also known as the HDV-BS that binds HDV RNP by interacting with the C-terminus of the large delta antigen. The second topology is the HBV assembly-competent topology, when the PreS1 and PreS2 domains, along with the TMDI and LCL, are all located inside the virion. In this case, the matrix domain (shown in green) located in the C-terminus of the PreS1/N-terminus of the PreS2 interacts with the HBV core protein (HBcAg) during the envelopment of the nucleocapsid. Also indicated is the N-terminus of the PreS1 domain that bears the sequences indispensable for the virions’ infectivity, including those involved in the binding to the NTCP. At the bottom of the figure is shown the small (S) envelope protein along with its numbering that is used in this study to indicate the positions of the DRMs in HBsAg.

**Fig 2:**
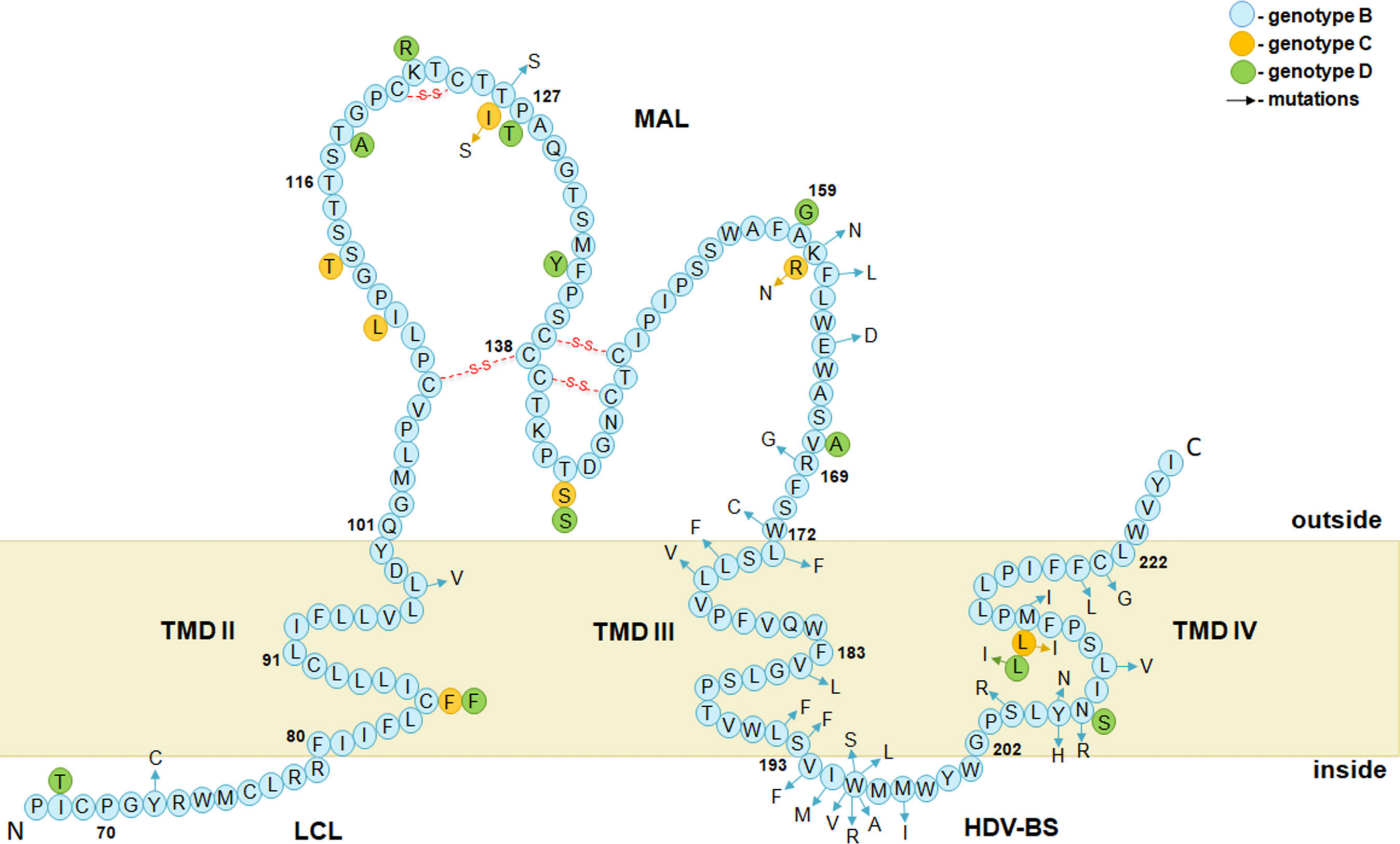
The DRMs selected for the study. The amino acid (aa) sequence of the small envelope protein from residue 67 in the LCL to the C-terminus bearing 30 DRMs selected for this study is shown. The aa differences between three different variants of HBsAg that belong to the HBV genotypes B, C, and D are indicated in different colors. The DRMs are depicted using the arrows. All the DRMs studied were single amino acid substitutions. Also indicated are the structural elements, such as LCL, TMDII, MAL, TMDIII, HDV-BS, and TMDIV, each of which contains at least one DRM. All TMDs’ sequences (including the boundaries of TMDs) were shown as embedded into the virion membrane and reflected only the location inside the membrane, but not the structural folding of the domains.

The human hepatitis delta virus (HDV) is a major human pathogen that can cause transient and chronic infections of the liver. HDV infection by itself can induce serious liver pathogenesis. HDV is the natural sub-viral agent of another very significant human pathogen, the human hepatitis B virus (HBV). While the HDV genome replication is independent of the HBV, HDV uses HBV envelope proteins (i.e., surface antigen, HBsAg) to assemble its own virions and to infect susceptible hepatocytes using the specific HBV receptor, which is the sodium taurocholate co-transporting polypeptide (NTCP). HDV takes only the envelope proteins from HBV, and all other factors needed for the completion of its life cycle HDV acquires from the host. Therefore, the natural mutations that occur during the HBV infection in the HBsAg have the potential to affect the yield of HDV virions and their infectivity (1–6). The drug resistance mutations (DRMs) that emerged in the sequence of HBsAg and were selected by anti-HBV treatments, which used FDA-approved nucleos(t)ide analogs, lamivudine (LMV), adefovir (ADF), telbivudine (TBV), entecavir (ETV), or tenofovir (TFV) (7–27), were the focus of this study. The primary sequence changes selected by the drug treatments occur in the open reading frame (ORF) of the HBV polymerase, leading to the corresponding drug resistance mutations in the overlapping ORF of HBsAg. DRMs were found in the large cytosolic loop (LCL), second transmembrane domain (TMDII), the major antigenic loop (MAL), third and fourth transmembrane domains (TMDs III and IV), and in the HDV-binding site (HDV-BS), which is a small cytosolic loop that consists of eight amino acids and is located between TMDIII and TMDIV (Fig. 1 and 2). A mutation in the polymerase ORF may create in HBsAg: (i) an amino acid (aa) change, (ii) no aa change, (iii) a STOP codon, or (iv) combinations of changes (7–27). We focused only on DRMs causing single aa changes in HBsAg, and also did not study HBV immune escape mutations (IEMs) in the MAL (13, 28, 29). We selected just one DRM in the LCL to be tested because previous work mutated the majority of the positions in the LCL and found that the generated mutants appeared to be permissive for HDV assembly and did not affect HDV infectivity (30). Only one DRM causing aa change in TMDII was reported (10, 16), seven DRMs were left in the MAL after excluding the IEMs (13, 28, 29), five DRMs - in TMDIII, eight - in HDV-BS, and eight - in TMDIV after omitting STOP codons and combinatorial changes selected by the drug treatments. These 30 DRMs (Table 1, Fig. 2) (7–27) were examined in this study. As reported previously, some DRMs could affect HBsAg production and secretion (11, 17, 20), HBV assembly (11, 17, 18), HBsAg antigenicity (11, 15, 21, 22), and HBV replication (11, 14, 17, 18, 22–25, 27). Among the DRMs presented in Table 1, there were some DRMs that appeared in previous publications. Thus, it was reported that E(164)D, I(195)M, W(196)S, and M(198)I reduced HBsAg antigenicity, while I(195)M and W(196)A/S/L/V/R reduced HBV replication (11, 15, 22–24, 27). Here and throughout the manuscript, the numbering for the DRMs' positions was given for the small envelope protein (Fig. 1 and 2). It, however, needs to be mentioned that another work evaluating the mutation E(164)A did not find any major changes in HBsAg antigenicity (31). In addition, it remains unclear how the above-mentioned DRMs in positions 195, 196, and 198, which all are located in the HDV-BS inside the virions (Fig. 1 and 2), could affect the HBsAg antigenicity, that is, its recognition by the anti-HBsAg antibodies, which only can bind HBsAg regions exposed on the outside surfaces of the viral particles.

**TABLE 1:** Drug resistance mutations (DRMs) in HBsAg selected for the analysis^*a*^

Mutation in S protein	Mutation in RT domain	Domain in HBsAg	Drug
Y(72)C	L(80)M	LCL	Lamivudine, tenofovir
L(98)V	S(106)C	TMD II	Tenofovir
T(126)S	D(134)E	MAL	Tenofovir
K(160)N	I(169)L		Lamivudine, entecavir
F(161)L	I(169)T		Entecavir
E(164)D	V(173)L		Lamivudine, telbivudine, entecavir, tenofovir
R(169)G	P(177)G		Tenofovir
W(172)C	A(181)S		Tenofovir
L(173)F	A(181)V		Lamivudine, adefovir, telbivudine, tenofovir
L(175)F	T(184)S	TMD III	Tenofovir
L(176)V	T(184)G		Entecavir
V(184)L	R(192)P		Tenofovir
L(192)F	A(200)V		Lamivudine, tenofovir
S(193)F	S(202)C		Lamivudine, entecavir
V(194)F	S(202)I	HDV-BS	Lamivudine, entecavir
I(195)M	M(204)V		Lamivudine, adefovir, telbivudine, entecavir, tenofovir
W(196)S	M(204)I		
W(196)L	M(204)I		
W(196)V	M(204)S		
W(196)A	M(204)S		
W(196)R	M(204)S		
M(198)I	V(207)L		Lamivudine, adefovir
S(204)R	S(213)T	TMD IV	
Y(206)H	V(214)A		Lamivudine, adefovir, tenofovir
Y(206)N	V(214)E		
S(207)R	Q(215)S		Adefovir, tenofovir
L(209)V	L(217)R		
L(213)I	F(221)Y		Tenofovir
F(220)L	L(229)V		Lamivudine, tenofovir
C(221)G	L(229)W		

^*a*^ The table displays the DRMs in HBsAg (7–27) investigated in this study. Shown are the DRMs in the small (S) envelope protein, corresponding mutations in the reverse transcriptase (RT) domain of the HBV polymerase, the corresponding domain in HBsAg bearing a particular DRM, and the anti-HBV drug(s), which selected for a particular DRM during the treatment. LCL, large cytosolic loop; TMDII, second transmembrane domain; MAL, major antigenic loop; TMDIII, third transmembrane domain; HDV-BS, HDV-binding site; TMDIV, fourth transmembrane domain.

Furthermore, the DRMs E(164)D and W(196)L/S were reported to reduce HBsAg secretion, while no such effect was observed for I(195)M. It was also observed that the DRMs W(196)L/S dramatically inhibited the egress of HDV virions, but the DRMs E(164)D and I(195)M apparently did not (20). Several natural combinations of mutations in HBsAg found in HBV genomes from actual patient were tested for their effect on HDV assembly and infectivity: F(20)S + P(120)S + E(164)D + L(173)F (mut 1), R(79)H + P(120)S + E(164)D + L(173)F + I(195)M + Y(206)F (mut 2), F (20)S + Δ102–111 + P(120)S + E(164)D + L(173)F (mut 3), and P(120)S + E(164)D + L(173)F + I(195)M (mut 4). Underlined are DRMs, but P(120)S is also the immune escape mutation in HBsAg. The delta mark represents the deletion of amino acids 102–111. These combinations did not reduce HBV replication. Mut 2 reduced HBsAg production and secretion. Mut 3 practically blocked HBsAg production/secretion. Mut 1, 2, and 4 reduced HBV assembly. No secreted HBV virions were found for mut 3. Mut 2 and 4 reduced HDV assembly, but mut 1 mediated a high yield of HDV virions. Only mut 3 blocked HDV assembly. Therefore, HDV infectivity was tested only for mut 1, 2, and 4, and no great impact on the infectivity of HDV virions was observed. However, the mutations were not tested individually, and 3 out of 4 above-mentioned combinations had additional changes (including one deletion) that were not DRMs. Thus, in that study, no information was acquired about the effects of individual DRMs on HDV assembly and infectivity (17).

The DRMs E(164)D, I(195)M, W(196)S, W(196)L, and L(173)F were included in our list of DRMs to be tested (Table 1). Overall, the vast majority of DRMs were never tested individually for their effect on HDV assembly, and no DRMs were tested individually for their impact on HDV infectivity. Thus, the mechanisms of how DRMs affect HDV properties, the course of HDV infection, and HDV pathogenesis remain very much unexplored. Our study, therefore, examined how 30 different DRMs in various regions of HBsAg (Table 1, Fig. 2) impacted HDV assembly and infectivity.

## RESULTS

### Influence of DRMs on the assembly of the virions of human hepatitis delta virus

Thirty different DRMs were analyzed. Each DRM was a single amino acid substitution, which has been naturally selected in HBV-infected individuals during the treatment with anti-HBV drugs, such as lamivudine, tenofovir, entecavir, adefovir, or telbivudine (7–27). All these DRMs are detailed in Table 1. We constructed vectors expressing HBV envelope proteins that contained either a single DRM or no DRMs (wild-type [wt] controls). The DRMs selected for the study were located in the large cytosolic loop (LCL), transmembrane domain II (TMDII), major antigenic loop (MAL), transmembrane domain III (TMDIII), HDV binding site (HDV-BS) that is a small cytosolic loop between the third and fourth transmembrane domains, and TMDIV of HBsAg (Fig. 1 and 2, Table 1). The above-mentioned structural elements of HBsAg are present on all three HBV envelope proteins, the large (L), middle (M), and small (S). To make the above-mentioned HBsAg-expressing vectors, we used previously described by us three constructs inducing the production of HBsAg, which belong to the genotypes B, C, or D of HBV, pNF-HBV-LMS-B2 (for the genotype B), pNF-HBV-LMS-C5 (for the genotype C), and pNF-HBV-LMS-D1 (for the genotype D) (32, 33). The constructs for the genotypes B and D express all three envelope proteins, L, M, and S, while pNF-HBV-LMS-C5 expresses only the L and S envelope proteins, because the sequence of the natural variant of the genotype C that was used to make this vector was an authentic M-minus mutant and did not express the M envelope protein. All these constructs were used by us previously to generate infectious virions of the hepatitis delta virus (32, 33). It needs to be noted that the expression of the L and S envelope proteins is sufficient to produce infectious virions of both HBV and HDV (1, 6, 32). The vectors expressing HBsAg of genotypes B and D did not bear any DRMs, and thus they served as the control vectors expressing wt HBV envelope proteins. The plasmid pNF-HBV-LMS-C5 was modified to contain S instead of R in position 204 (as mentioned above, the numbering is for the small envelope protein), thereby eliminating the DRM S(204)R present in the natural variant of genotype C used to make this plasmid. The modified plasmid was named pNF-HBV-LMS-C5wt, and it served as a wt HBsAg-producing control plasmid for all DRM-bearing HBsAg-expressing vectors that were made in the context of the HBV genotype C.

**Fig 3:**
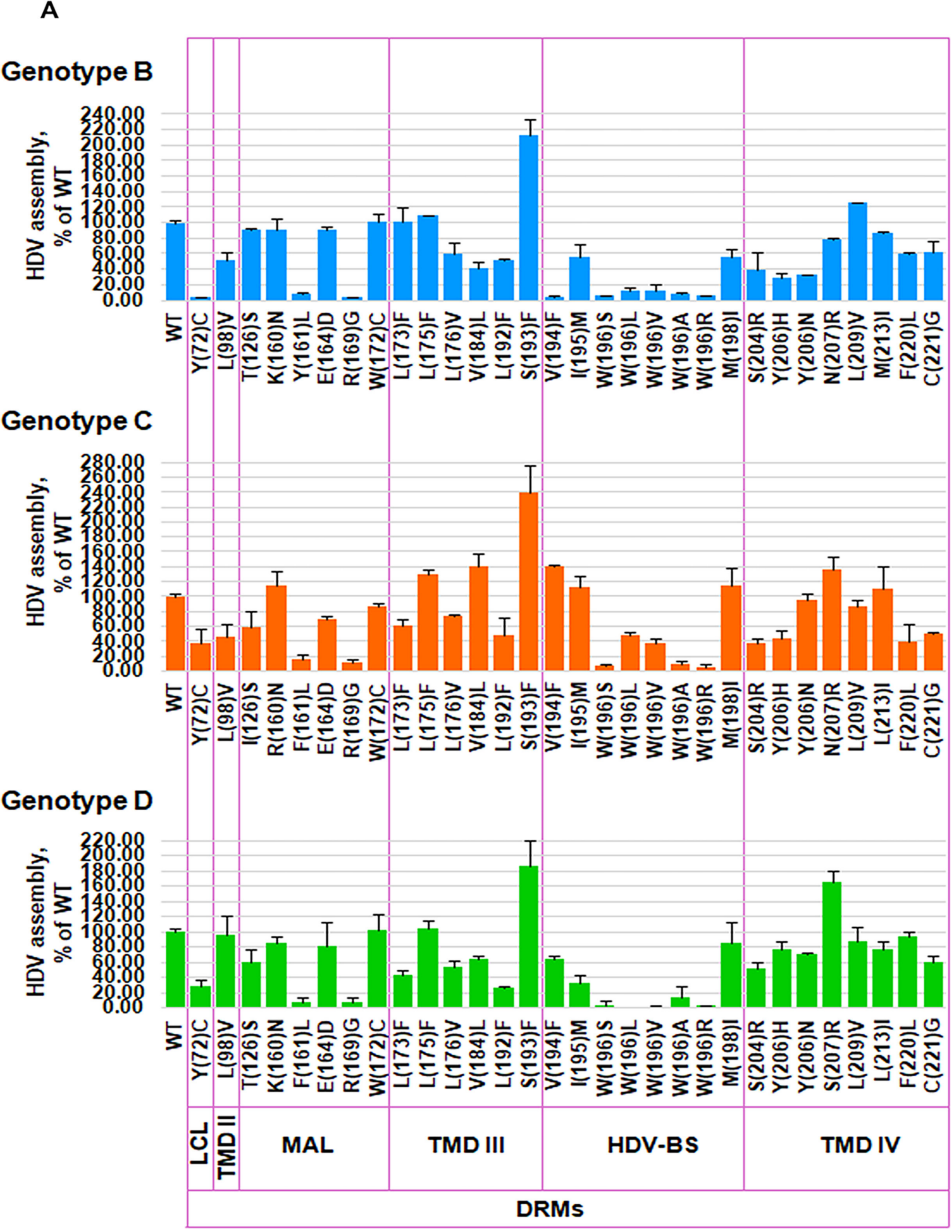

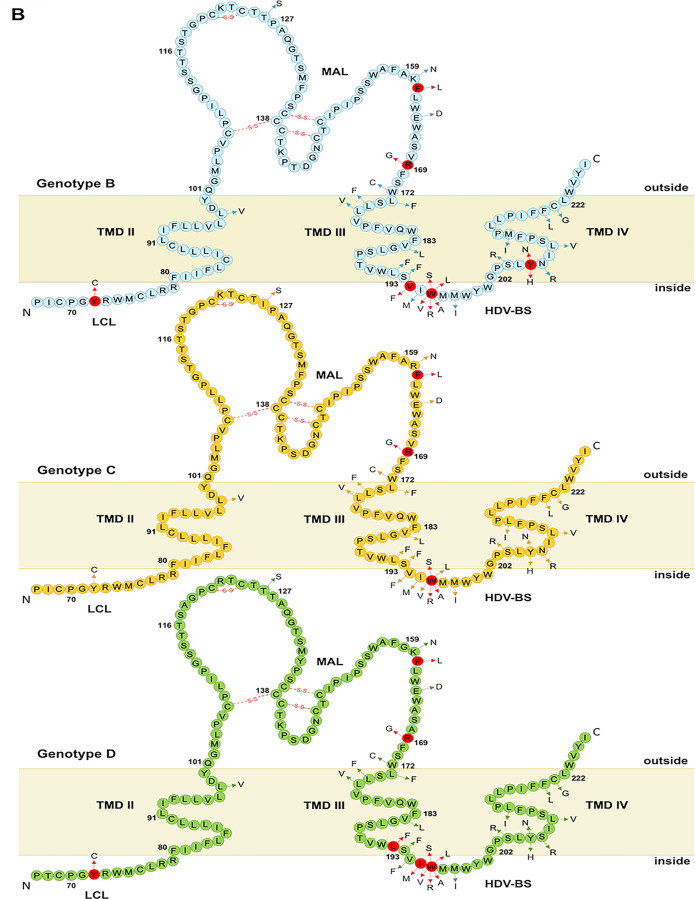
Influence of DRMs in HBsAg on the assembly of HDV. (**A**) Levels of secreted HDV virions. The analysis of the HDV assembly is detailed in the Materials and Methods. The levels of secreted HDV virions in the media, which were collected from Huh7 cells co-transfected with the construct pSVLD3 initiating HDV genome replication and a plasmid expressing HBsAg of a particular HBV genotype with or without DRM, are expressed as the bars reflecting the percentage relative to the HDV assembly levels observed when wt HBsAg (not bearing DRMs) was used. The HDV assembly levels found when wt HBsAg was used for the formation/secretion of HDV virions were used as the 100% value. Each diagram presents the results for one of the tested HBV genotypes, B, C, or D. The wt and DRMs are indicated at the bottom of the diagrams. The corresponding structural elements of HBsAg bearing the tested DRMs are indicated underneath the diagrams. A linear mixed-effects model was used to analyze the difference in assembly levels between wt and DRMs, with DRM as a fixed effect and experiment as a random effect. The effects of the DRMs that showed 3-fold or greater decrease in the assembly levels compared to wt were statistically significant at the 5% significance level (*P*-value < 0.05, for each comparison between wt and each DRM). The only mutant L(192)F in the context of the genotype D of HBV had a *P*-value = 0.073, which was statistically significant at the 10% significance level. (**B**) Locations of the DRMs that affected the levels of HDV assembly. Shown are the amino acid sequences of the region of the S envelope protein (from position 67 to the C-terminus of the S) for the HBsAg variants of the HBV genotypes B, C, and D that were used in the study. The amino acid chains reflect different structural elements and the domains of the S envelope protein located inside or outside the virions, including TMDs embedded in the virion’s membrane. The DRMs that decreased the levels of the HDV assembly (as shown in panel **A**) by 3-fold or more are indicated in red.

The above-described HBsAg-expressing vectors with and without DRMs were then used for producing HDV virions. In each case, Huh7 cells were co-transfected (in a 1:1 ratio) with the plasmid pSVLD3 initiating HDV genome replication and one HBsAg-expressing construct (with or without a DRM), and the levels of secreted HDV were measured at day 9 post-transfection using RT-qPCR as described in the Materials and Methods section (32–34). The outcomes of this analysis are shown in Fig. 3.

Only DRMs located in the HDV-BS that spans amino acid positions 194–201 in the S envelope protein could affect HDV assembly directly by influencing the interactions of the large delta antigen with the HDV-BS in HBsAg (1, 4, 30) (Fig. 1 and 2; Table 1). The DRMs located in other structural elements of the S domain of HBsAg could influence the formation of HDV virions only via indirect mechanisms. All tested DRMs were permissive for the assembly of HDV virions. The 17/30 DRMs had no considerable effect on HDV assembly for the HBV genotypes B, C, and D as compared to the corresponding wt HBsAg. They were L(98)V, T/I(126)S, K/R(160)N, E(164)D, W(172)C, L(173)F, L(175)F, L(176)V, V(184)L, S(193)F, M(198)I, S(204)R, N/S(207)R, L(209)V, M/L(213)I, F(220)L, and C(221)G. These DRMs facilitated the yield of HDV virions within the range from 37.99% to 238.49% as compared to the wt HBsAg (Fig. 3), while none of the observed effects on the levels of secreted HDV virions exceeded a 3-fold difference as compared to the levels of secreted HDV facilitated by wt HBsAg that were used as 100% values. One DRM S(193)F, however, should be noted for its ability to increase the levels of secreted HDV by ~187%–238% on the background of all three tested HBV genotypes.

The remaining 13 analyzed DRMs affected the yield of secreted HDV virions by 3-fold or more (Fig. 3). All the effects observed for these DRMs reflected reductions of HDV assembly levels. Some of the observed effects were HBV genotype-specific and differed substantially between the tested HBV genotypes. Thus, the only DRM located in the LCL Y(72)C reduced HDV assembly by ~29.3-fold for the HBV genotype B, and only by ~3.6-fold for the genotype D, while the effect of this DRM for the genotype C was less than 3-fold. The Y/F(161)L located in the MAL decreased HDV assembly for all three HBV genotypes in the range from ~6.0-fold to 14.2-fold. Another DRM R(169)G that also resided in the MAL reduced the levels of secreted HDV virions by ~8.9-fold to 26.8-fold. Out of the DRMs located in TMDIII, only L(192)F reduced HDV assembly by ~3.7-fold and only for the genotype D of HBV (Fig. 3). As anticipated, the most impactful were DRMs located in the HDV-BS. Surprisingly, the V(194)F greatly reduced HDV assembly almost by 22-fold only for the genotype B of HBV, while the same DRM did not have a major influence on the levels of secreted HDV in the context of the genotypes C and D. The DRM I(195)M reduced the levels of secreted HDV virions by about 3-fold only for the genotype D. The DRM W(196)S greatly decreased HDV assembly within the range of ~11.7-fold to 25.4-fold for all three HBV genotypes B, C, and D. The DRMs W(196)L and W(196)V had similar effects and very much reduced the levels of secreted HDV virions for the genotypes B (~8.2-fold to 8.5-fold) and D (~72.5-fold to 139.7-fold), but did not have a great effect on the HDV assembly for the genotype C. The next two DRMs in the HDV-BS, W(196)A and W(196)R, decreased the yield of HDV virions for all three HBV genotypes in the range from 7.0-fold to 30.1-fold. Thus, different amino acid substitutions at the same position in HDV-BS could lead to somewhat different effects. As mentioned above, the DRM M(198)I had no great effect on HDV assembly. Our data are consistent with the previous study that reported the ability of the W(196)S/L to considerably reduce the levels of secreted HDV virions, while it did not find a dramatic reduction of the HDV assembly for the I(195)M and E(164)D mutants. Although this work has been done using genotype A of HBV (20). Similar to the DRMs in the TMDIII, the DRMs in the TMDIV were well tolerated in terms of the HDV assembly, and only Y(206)H and Y(206)N reduced the yield of secreted HDV by about 3-fold only in the context of the genotype B (Fig. 3).

**Fig 4:**
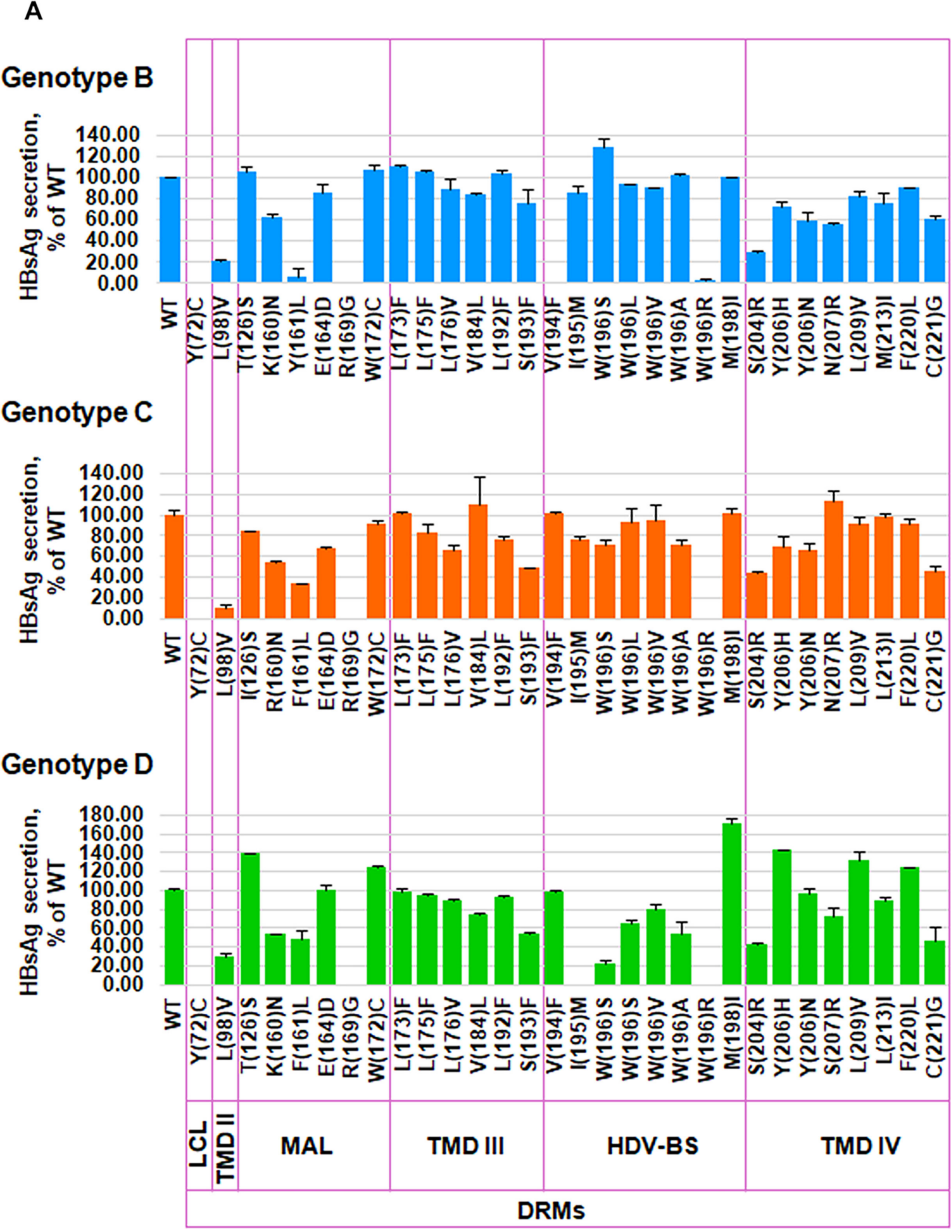

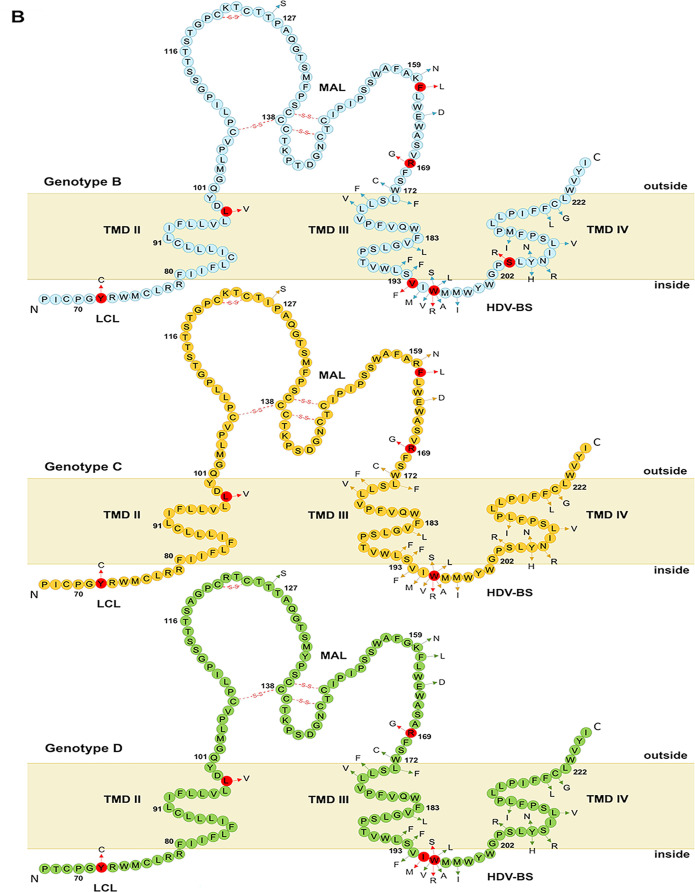
Influence of DRMs on the secretion of HBsAg. (**A**) Levels of secreted HBsAg. The analysis of the levels of secreted HBsAg using ELISA is described in the Materials and Methods. The HBsAg levels in the media harvested from Huh7 cells co-transfected with the plasmid pSVLD3 starting HDV replication and a vector expressing HBsAg variant of a particular HBV genotype (bearing or not bearing a DRM) are shown as the bars reflecting the percentage relative to the HBsAg secretion levels measured for wt HBsAg (not bearing DRMs), which were used as 100% levels. Each diagram presents the results obtained for one of the tested HBV genotypes, B, C, or D. The wt and DRMs are shown at the bottom of each diagram. At the bottom of the figure are also indicated the corresponding structural elements of HBsAg containing the analyzed DRMs. A linear mixed-effects model was used to analyze the difference in HBsAg secretion levels between wt and DRMs, with DRM as a fixed effect and experiment as a random effect. The effects of the DRMs that showed 3-fold or greater decrease in HBsAg secretion levels compared to wt were statistically significant at the 5% significance level (*P*-value < 0.05, for each comparison between wt and each DRM). (**B**) Locations of the DRMs that impacted the levels of HBsAg secretion. The sequences of the region of the S envelope protein spanning position 67 to the C-terminus for the HBsAg variants of the tested HBV genotypes B, C, and D are shown. The amino acid chains reflect different structural elements of the S protein located inside or outside the virions. The DRMs that decreased the levels of the HBsAg secretion (as shown in panel A) by ≥3-fold are indicated in red color.

We next assayed the yield of secreted HBsAg using ELISA (Abbexa). We have, however, considered that previously published studies reported that there was frequently no direct correlation between the measured levels of secreted HBV envelope proteins and the yield of HBsAg-coated HDV virions, because in the infection settings, and in the settings of the production of HDV virions using a cell culture, the HBsAg is made in excessive amounts, and most of HBsAg gets secreted as empty sub-viral particles (SVPs), which bear inside no HDV RNA genome in the complex with delta antigens that form the HDV ribonucleoprotein (or HDV RNP), and only a minor fraction of HBsAg is egressed in the context of HBsAg-coated HDV virions. Furthermore, the efficiency of HDV assembly depends in large part on the functional properties of the HBV envelope proteins rather than just on the efficiency of HBsAg production (31, 32, 35, 36). Therefore, it should be noted that the assayed yield of secreted HBsAg is expected mostly to reflect the levels of SVPs and not necessarily the yield of secreted HDV virions. In addition, some mutations in certain HBsAg domains exposed on the outside of the virions can interfere with the ability of the antibodies to recognize and bind HBsAg-enveloped HDV virions. Having considered the above arguments, we still reasonably expected that at least for the DRMs, which are not located on the outside of the virions, we should be able to obtain useful information allowing us to conclude whether the amounts of secreted HBsAg could contribute to substantially decreased levels of secreted HDV virions, as mentioned above. The results of the measurements of the levels of secreted HBsAg are presented in Fig. 4. For the DRM Y(72)C located in the LCL, which is inside the virion (Fig. 1 and 2) and thus should not affect the binding of HBsAg by anti-HBsAg antibodies, HBsAg in media was undetectable for all three HBV genotypes (Fig. 4), while the measurable levels of HDV virions were observed for all three tested HBV genotypes (Fig. 3). This is not surprising because RT-qPCR is a considerably more sensitive assay than ELISA, and it just illustrates the fact that HDV uses small amounts of produced HBsAg for its assembly. Furthermore, undetectable HBsAg (Fig. 4) correlates with a very low yield of HDV virions for the HBV genotype B (3.4% as compared to the wt HBsAg), and reduced levels of HDV virions for the two other HBV genotypes, including the genotype D, for which the reduction of the yield of secreted HDV was a little more than 3-fold (Fig. 3). These results also demonstrated the HBV genotype-specific differences attributed to the capacity of HBsAg of different HBV genotypes bearing the same DRM to facilitate the assembly of HDV. The reduced yield of HBsAg by 3.4-fold to 9.7-fold for DRM L(98)V in TMDII (Fig. 4), which should not interfere with the antibody-virus interactions as well (Fig. 1 and 2), did not result in a considerable decrease of HDV assembly for all three HBV genotypes (Fig. 3). This finding further illustrates the point that the efficiency of HDV assembly often does not correlate with the levels of secreted HBsAg. The decrease of HBsAg levels by 14.6-fold and 3-fold for the genotypes B and C, respectively, for DRM Y/F(161)L in the MAL (Fig. 4) was consistent with reduced HDV assembly levels (Fig. 3). However, because HDV assembly was decreased for all three HBV genotypes, and the degree of its reduction was similar to the reduced yield of HBsAg only for the genotype B, but it was considerably more decreased by about 6-fold to 14.2-fold for the genotypes C and D (as compared to the corresponding HBsAg levels), it is reasonable to think that at least for the genotypes C and D, this DRM altered the properties of HBsAg, which contributed to diminished HDV assembly. Furthermore, in this case, we should also take into account that position 161 was in the MAL and it was not reported previously as critical for the antigenicity of HBsAg (31), and thus the ELISA data for this mutant are expected to reflect HBsAg yield, and not the altered recognition by anti-HBsAg antibodies. For another DRM R(169)G in the MAL, the ELISA did not detect secreted HBsAg (Fig. 4), which was consistent with the greatly reduced levels of HDV assembly by 8.9-fold to 26.7-fold (Fig. 3). We also considered that position 169 in the MAL, similarly to position 161, was reported as the one that was not important for HBsAg antigenicity (31). Other tested DRMs located in the MAL did not have a considerable effect on the levels of secreted HBsAg. For the DRMs in the TMDIII, we did not observe any considerable decrease of HBsAg levels (Fig. 4), which suggested that the reduction of the yield of secreted HDV virions for the DRM L(192)F for the genotype D of HBV by about 3-fold (Fig. 3) reflected the alteration of the functional properties of HBsAg bearing this DRM. Next were the DRMs located in the HDV-BS, which could not affect the HDV-antibody interactions (Fig. 1 and 2). Only for V(194)F in the context of genotype B, the undetectable HBsAg levels were consistent with the 21.7-fold reduction of HDV assembly. Interestingly, for the other two HBV genotypes C and D, practically unaffected levels of secreted HBsAg (Fig. 4) correlated with considerable levels of HDV assembly (Fig. 3). For the I(195)M, undetectable HBsAg for the genotype D of HBV (Fig. 4) was consistent with the reduction of HDV assembly by 3-fold (Fig. 3). For the DRM W(196)R, the HBsAg yield was greatly decreased by about 29-fold for the genotype B, and for the other two HBV genotypes HBsAg was undetectable (Fig. 4), which very much correlated with greatly reduced HDV assembly levels in the 16-fold to 30-fold range for all three HBV genotypes tested. In addition, for the W(196)S, substantially reduced yield of secreted HBsAg by ~4.4-fold for the genotype D correlated with greatly reduced HDV assembly by 25.4-fold. Interestingly, for the same DRM, the HBsAg secretion was not considerably affected for the HBV genotypes B and C, while the corresponding HDV assembly levels were dramatically reduced (by 11.7-fold to 14.9-fold) just as it was found for the genotype D (Fig. 3). Thus, similarly for the above-mentioned Y/F(161)L case, at least for the genotypes B and C, the alteration of the functional properties of HBsAg by W(196)S was the likely reason mediating reduced HDV assembly. It needs to be noted that the altered HBsAg properties also could have contributed to reduced HDV assembly by the DRM W(196)S in the context of the HBV genotype D, because the reduction of the HDV assembly in this case was about 5.8 times more pronounced than the corresponding reduction of HBsAg levels (Fig. 3 and 4). For the other DRMs in HDV-BS, for which we observed a great reduction in HDV assembly levels (Fig. 3), these effects reflected the alteration of the interactions between the HBsAg and large delta antigen during the formation of HDV virions, because these DRMs did not cause any considerable reduction of the yield of secreted HBsAg (Fig. 4). There was no considerable reduction in HBsAg levels for the DRMs in TMDIV (Fig. 4), except for the S(204)R in the context of the HBV genotype B (by ~3.3-fold), which, however, did not cause a substantial reduction of the HDV assembly levels. At the same time, the decrease in the yield of secreted HDV virions for DRMs Y(206)H and Y(206)N by about 3-fold for the genotype B of HBV (Fig. 3) should also be attributed to the changes in the functional properties of HBsAg, because there was no great decrease in the levels of secreted HBsAg (Fig. 4).

In summary, in 5/13 cases (i.e., Y(72)C, R(169)G, V(194)F, I(195)M, and W(196)R), the decreased yield of secreted HBsAg most likely was the considerable contributing factor for the reduced HDV assembly in the majority of the situations. In other 6/13 cases, the primary reason for the decreased yield of secreted HDV virions was most likely the alteration of HBsAg functional properties resulting (i) in impaired interactions between HBV envelope proteins and large delta antigen, which acted in the context of HDV RNP (i.e., W(196)L, W(196)V, and W(196)A that were located in the HDV-BS), or (ii) in other defects affecting indirectly either the formation of HDV virions and/or their egress (i.e., L(192)F, Y(206)H, and Y(206)N that were located in TMDs III and IV and not in the HDV-BS, and thus were not directly involved in the interactions between HBsAg and the large delta antigen of HDV RNP). In 2/13 cases, for the DRMs Y/F(161)L and W(196)S, both the decreased levels of secreted HBsAg and altered HBsAg properties likely contributed to reduced HDV assembly (depending on the HBV genotype).

### DRMs located in various domains of HBV envelope proteins affect the infectivity of HDV virions

The tested DRMs were located in the LCL, TMDII, MAL, TMDIII, HDV-BS, and TMDIV (Fig. 1 and 2 , Table 1). Only DRMs in the MAL are exposed on the outside of the virions, and thus potentially can affect the attachment and entry of HDV virions to susceptible hepatocytes. The DRMs in the MAL can also potentially influence the post-entry intracellular trafficking of the virions. These DRMs, in theory, could indirectly (because they are not involved in direct interactions between the large delta antigen and HBsAg) affect the uncoating process as well. The uncoating occurs some time after the entry, when the HDV RNP loses the envelope and gets disassembled from the HBV envelope proteins. The DRMs located in the other above-mentioned domains are not positioned on the outer surface of the virions. They are therefore unlikely to affect the process of the virions’ attachment and entry, and also to influence the intracellular trafficking of HDV virions. They, however, could participate in the uncoating process. During the uncoating, the DRMs located in the HDV-BS could directly impact the disassembly process, while DRMs in the other locations may influence the uncoating indirectly. Altering any of the above-mentioned steps of the infection (i.e., attachment and entry, post-entry intracellular trafficking, and uncoating) will affect the overall infectivity of the HDV virions.

For *in vitro* infection with HDV, we used the HepG2-NTCP cells bearing the HBV receptor, human NTCP (3), and which can be infected with either HBV or with HDV coated with the HBV envelope proteins (37). For our infection experiments, we used HDV enveloped with HBsAg of the genotype D of HBV, which contained or did not contain a DRM. In the settings of our analysis, each tested DRM was present on all the envelope proteins, the L, M, and S. As the control, we used HDV virions coated only with the S envelope protein, which are known to be not infectious (32, 33). The HepG2-NTCP cells infected with HDV have been analyzed at day 8 after the infection.

We have analyzed two parameters of the infectivity of HDV virions. The first parameter that we determined was the levels of the accumulation of the infection-generated HDV genomic RNA (HDV G RNA) in the infected cells (32, 33). For comparison purposes, the intracellular HDV G RNA accumulation levels were presented as the percentage relatively to the levels of HDV G RNA that were observed for the HDV virions enveloped with wt HBsAg not bearing DRMs, which were used as 100% value. All *in vitro* HDV infections conducted to quantify the accumulation of HDV G RNA in the infected HepG2-NTCP cells were performed using the multiplicity of infection (MOI) of 70 HDV genome equivalents per cell (GE/cell). The MOI reflects the number of the HDV GE/cell used in the inoculum during the infection. Second, by employing the immunofluorescence (IF) and the staining of the cells for the intracellular delta antigens, production of which resulted from HDV infection and replication of the HDV genomes in infected cells, we examined the percentage (fraction) of the HDV-infected cells (32, 33). For the subsequent comparative analysis, the data reflecting the fraction of the HDV-infected cells were expressed as the percentage relatively to the fraction of the infected cells found for the HDV virions enveloped with wt HBsAg not bearing DRMs, which was used as a 100% value. The infections for the analysis of the percentage of HDV-infected cells have been done using the MOI of 100 (HDV GE/cell).

**Fig 5:**
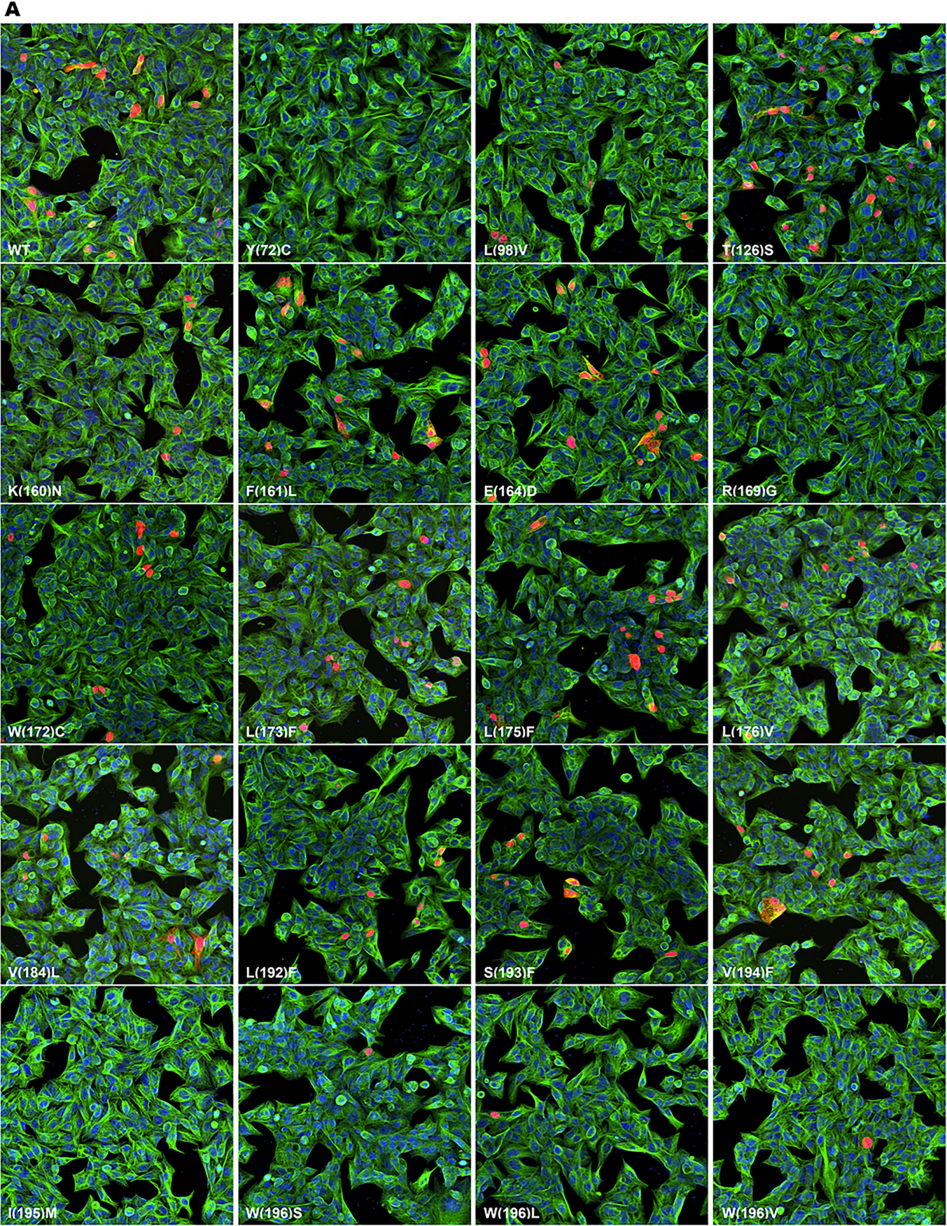

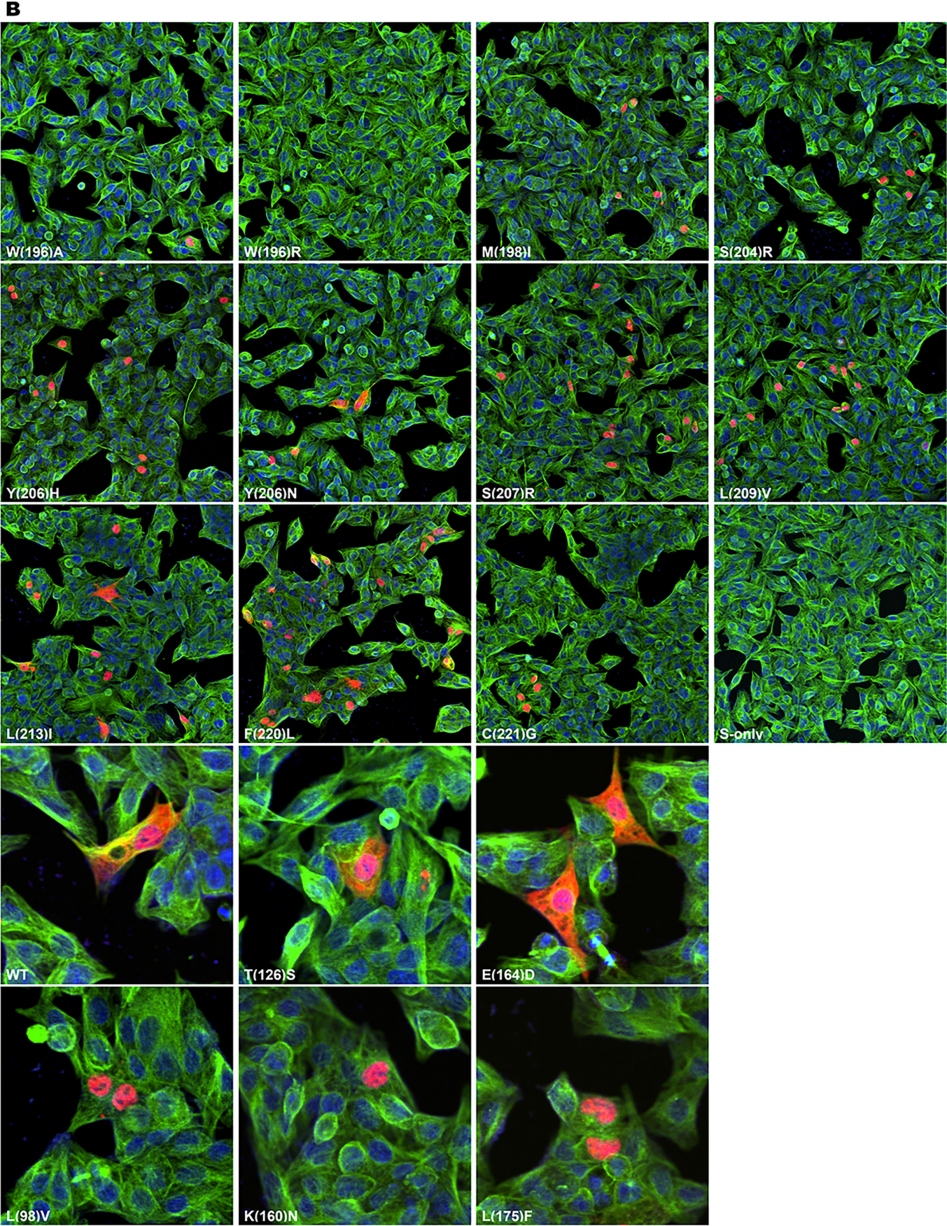
HepG2-NTCP cells infected with HDV virions enveloped with HBsAg containing DRMs. The images of the HepG2-NTCP cells infected with HDV virions coated with different variants of HBsAg of the genotype D of HBV, which contain or do not contain DRMs, are shown. The type of HBV envelope proteins with or without DRM, which coated the HDV virions in the corresponding experiment, is indicated on each image. The WT stands for the HBsAg not containing DRMs (i.e., first control). The second control was non-infectious HDV virions enveloped with the wt S envelope protein only (32) (indicated as S-only). The HepG2-NTCP cells were examined at day 8 post-infection with HDV using the IF as described in the Materials and Methods. The green staining is for the cellular alpha-tubulin, the red staining is for the intracellular delta antigens generated by the HDV infection (which indicates the HDV-infected cells), and the blue staining is for nuclear DNA (DAPI, 4′,6′-diamidino-2-phenylindole). The panel **A **displays the results for the WT, and the following DRMs: Y(72)C, L(98)V, T(126)S, K(160)N, F(161)L, E(164)D, R(169)G, W(172)C, L(173)F, L(175)F, L(176)V, V(184)L, L(192)F, S(193)F, V(194)F, I(195)M, W(196)S, W(196)L, and W(196)V. The panel B reflects the results for the following DRMs: W(196)A, W(196)R, M(198)I, S(204)R, Y(206)H, Y(206)N, S(207)R, L(209)V, L(213)N, F(220)L, C(221)G, and for the S-only control. In the bottom two rows of panel **B**, the examples of the two major patterns of the subcellular localization of the delta antigens in the infected cells are also shown. The pattern that includes the delta antigens in the nucleoplasm (with nucleoli exclusion) along with the delta antigens in the cytoplasm is the less frequently observed one, and it is represented in the figure by the images for the WT and DRMs T(126)S and E(164)D. The most frequently seen pattern of the delta antigens localized in the nucleoplasm (with nucleoli exclusion) is represented by the images for the DRMs L(98)V, K(160)N, and L(175)F.

Figure 5A and B shows the IF images of the HDV-infected HepG2-NTCP cells, which were obtained for the infection with HDV coated with wt HBsAg or with HDV virions enveloped with DRM-bearing HBsAg. As expected (32, 38), we observed two patterns of the intracellular localization of delta antigens: (i) diffused nucleoplasmic staining with nucleoli exclusion (the examples are: L(98)V, K(160)N, and L(175)F), and (ii) cytoplasmic staining along with nucleoplasmic localization (the examples are as follows: WT, T(126)S, and E(164)D) (Fig. 5B). The former is the most frequent pattern of the intracellular distribution of delta antigens in the context of the HDV infection/replication, while the latter occurs much less frequently (32, 38). We previously observed both of the above-mentioned patterns in the HDV-infected primary human hepatocytes (32, 38).

**Fig 6:**
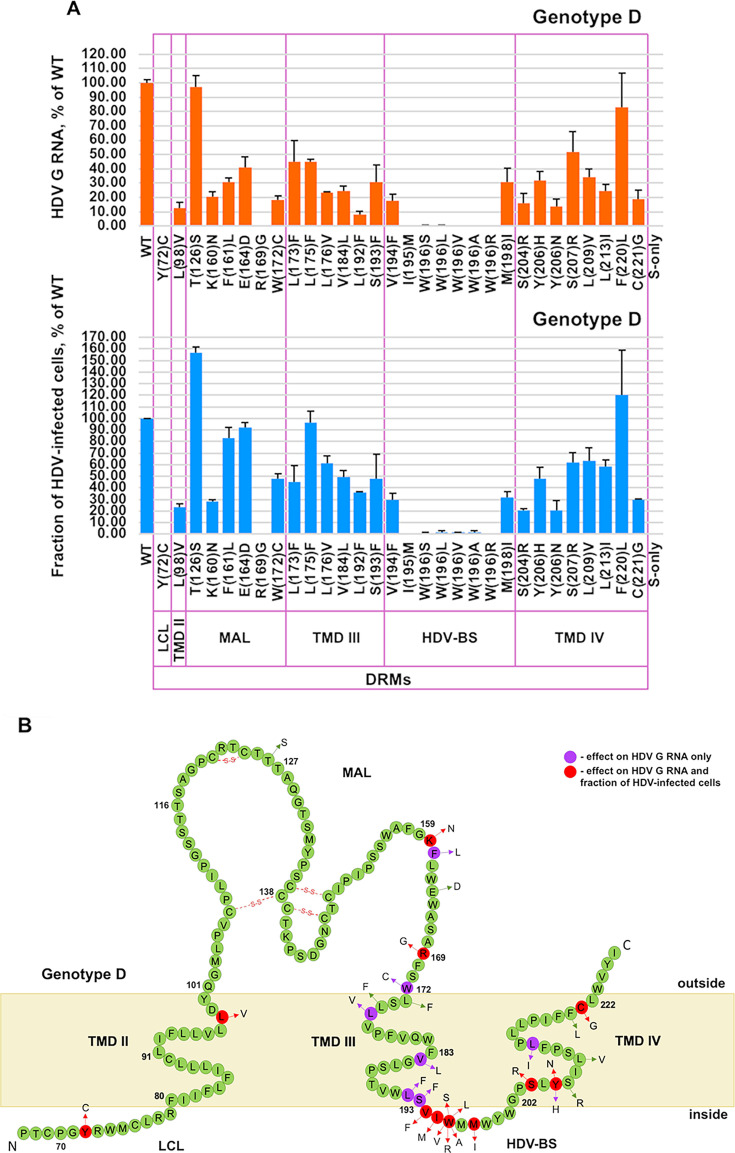
Analysis of the infectivity of HDV virions enveloped with HBsAg bearing DRMs. Two parameters of the infectivity of HDV virions coated with HBsAg variants (with and without DRMs) of the HBV genotype D were examined. The first parameter was the accumulation levels of HDV genomic RNA (HDV G RNA) in the infected cells. The second parameter was the fraction of the HDV-infected cells. Both parameters are described in detail in the text. (A) The levels of HDV G RNA in the infected cells (top diagram) and fraction of the infected cells (bottom diagram) are shown as the bars displaying the percentage value of each parameter (i.e., levels of HDV G RNA or the fraction of the infected cells, respectively) relatively to the levels of the corresponding parameter, which were obtained for the HDV virions coated with wt HBsAg (not bearing DRMs) and which were used as 100% values. The experiments to determine the levels of HDV G RNA in the infected cells were done using the MOI of 70 (i.e., 70 HDV GE/cell in the inoculum during the infection). The experiments to determine the fraction of the HDV-infected cells were conducted using the MOI of 100. The wt, DRMs, and S-only are indicated at the bottom of the diagrams. At the bottom of panel A, the structural elements of HBsAg bearing the corresponding DRMs are also shown. An independent two-sample t-test was conducted to compare DRMs that showed 3-fold or greater decrease in the HDV G RNA values compared to wt (group 1) and DRMs that showed less than 3-fold decrease in HDV G RNA values compared to wt (group 2). There was a significant difference between groups 1 and 2 (*P*-value = 0.000148). In addition, an independent two-sample t-test was conducted to compare DRMs that showed ≥3-fold decrease in the fraction of the HDV-infected cells values compared to wt (group 1) and DRMs that showed less than 3-fold decrease in the fraction of the HDV-infected cells values compared to wt (group 2). There was a significant difference between groups 1 and 2 (*P*-value < 0.0001). (B) The DRMs that impacted either the levels of HDV G RNA alone (indicated in purple) or both the levels of HDV G RNA and the fraction of HDV-infected cells (indicated in red) are shown within the aa sequence of the S envelope protein (position 67 to the C-terminus of the S), which is presented as the aa chain bearing the part of the LCL along with TMDII, MAL, TMDIII, HDV-BS, and TMDIV. The DRMs affecting the levels of HDV G RNA and the fraction of HDV-infected cells are also indicated at the same time using a red arrow, and the DRMs that only affected the levels of HDV G RNA but not the fraction of the infected cells are indicated using a purple arrow, which was necessary because, on more than one occasion, we studied more than one DRM for the same position in HBsAg. In addition, it needs to be noted that only in one situation, for position Y206, it is marked as the red circle, indicating that the DRM Y(206)N affected both above-mentioned parameters of HDV infectivity, and this effect is also shown with the red arrow. While at the same position, Y206 in HBsAg, the DRM Y(206)H affected only the levels of HDV G RNA, which is indicated by the purple arrow.

The outcomes of the analysis of the HDV infectivity (the relative levels of intracellular HDV G RNA and the fraction of HDV-infected cells) are shown in Fig. 6. None of the DRMs considerably increased the infectivity of HDV virions. Only 7/30 DRMs had no considerable (i.e., ≥3-fold) effect on the infectivity of HDV. They were the T(126)S (MAL), E(164)D (MAL), L(173)F (TMDIII), L(175)F (TMDIII), S(207)R (TMDIV), L(209)V (TMDIV), and F(220)L (TMDIV). The majority of tested DRMs considerably reduced HDV infectivity (i.e., either the levels of HDV G RNA in infected cells only or both the accumulation of intracellular HDV G RNA and the fraction of the infected cells) by ≥3-fold as compared to the HDV virions coated with wt HBsAg (Fig. 6).

Four DRMs resulted in the generation of non-infectious HDV virions: Y(72)C (LCL), R(169)G (MAL), I(195)M (HDV-BS), and W(196)R (HDV-BS). For them, no HDV-infected cells were found, and the levels of HDV G RNA were of 0% (Fig. 5 and 6). For the DRMs I(195)M and W(196)R, which both belong to the HDV-binding site that is involved in the binding of the HBsAg to HDV RNP (1, 4, 30, 39), it could be reasonable to expect that these two DRMs severely affected the interactions between the large delta antigen and HDV-BS in the HBsAg, leading to the inability of the HDV virions to infect the susceptible cells. Furthermore, these DRMs resulted in undetectable HBsAg by ELISA indicating the defects in the HBsAg synthesis and/or secretion (Fig. 4), and they also reduced the yield of secreted HDV virions by about 3-fold for I(195)M, and by about 30-fold for W(196)R indicating possible defects in virions formation and/or secretion as well (Fig. 3). The altered interactions between HBsAg and HDV RNP could potentially dysregulate both the assembly of HDV virions and also the post-entry disassembly of HDV RNP from HBsAg inside the infected cells (i.e., uncoating of the virions). The Y(72)C and R(169)G also resulted in undetectable HBsAg by ELISA (Fig. 4), and considerably decreased levels of secreted HDV virions (Fig. 3), indicating an altered assembly process, which, as mentioned above, could also lead to dysregulated uncoating of HDV virions. However, none of these two DRMs was located in the HDV-BS (Fig. 1 and 2), and thus cannot be involved directly in the interaction of large delta antigen and HBsAg. The DRM Y(72)C is located in the large cytosolic loop of HBsAg (Fig. 1 and 2), which is inside the virion and, as mentioned above, is not involved directly in the interactions between HBsAg and HDV RNP, and in the attachment/entry or the post-entry trafficking as well. While it may potentially take part in the uncoating process as suggested above, the mechanism of how the DRM Y(72)C renders the HDV virions not infectious is not immediately apparent. The DRM R(169)G located in the MAL, in theory, can not only affect the intracellular uncoating as indicated above, but may also impair the functioning of the MAL during the attachment/entry and during the intracellular post-entry trafficking. However, the exact mechanism of this DRM’s involvement in the altering of the infection process remains not completely elucidated yet, and therefore further analysis more directly examining different steps of the HDV infection process that are facilitated by the HBV envelope proteins, including the attachment, entry, post-entry intracellular trafficking, and the uncoating of the virions (when the viral cargo gets disassembled from the envelope), is warranted in the follow-up studies.

Other DRMs located in the small cytosolic loop of HBsAg or HDV-BS, 194-VIWMMWYW-201 (Fig. 1 and 2) (39) also considerably diminished HDV infectivity, which was evident by markedly low (i) levels of HDV G RNA and (ii) the fraction of the infected cells (Fig. 5 and 6). Out of these DRMs, the lesser impact on the infectivity was mediated by V(194)F, which reduced the levels of HDV G RNA by 5.6-fold, and the fraction of the infected cells by 3.4-fold, and M(198)I that decreased both parameters used to evaluate the HDV infectivity by about 3-fold. The rest of the DRMs in HDV-BS were located at position 196 (Table 1), and they resulted in greatly diminished accumulation of HDV G RNA by 335.6-fold to 437.1-fold, and the fraction of the infected cells was also very much reduced by 56.4-fold to 108.8-fold (Fig. 6). The amino acids of the small cytosolic loop of HBsAg (i) were shown to be critical for HDV assembly (39), and (ii) apparently were not important for HBV assembly and infectivity (30). In addition, we previously reported that a natural HBsAg mutant bearing the substitution Y(200)F in the HDV-BS substantially reduced HDV infectivity (32). Therefore, it became apparent that HDV-BS could play a critical role likely in influencing the intracellular post-entry uncoating of HDV virions by regulating the disengagement of the HDV RNP from HBsAg. At the same time, HDV-BS, being inside the virion, is not likely to be involved in the attachment/entry and the post-entry trafficking. Therefore, a considerable reduction of the HDV infectivity for the DRMs situated in the HDV-BS was not unexpected.

The results obtained for the DRMs located in the transmembrane domains were very surprising. There were DRMs in each of the three tested TMDs (i.e., TMDII, TMDIII, and TMDIV), which greatly reduced the infectivity of HDV virions (Fig. 5 and 6). We thus demonstrated that the sequences of TMDs II, III, and IV were actually critically important for the regulation of HDV virions’ infectivity. There were no previous reports implicating the TMDII, TMDIII, and TMDIV in influencing the infectivity of the virions. In more detail, the only tested DRM in TMDII, L(98)V, greatly reduced both the levels of intracellular HDV G RNA and the fraction of the infected cells, by 7.8-fold and 4.3-fold, respectively (Fig. 6). The L(176)V and V(184)L located in TMDIII diminished only the levels of HDV G RNA by a little greater than 4-fold without a great impact on the fraction of the infected cells, which was consistent with TMDIII being embedded into the virion’s membrane and not being exposed on the outside of the virion, and therefore TMDIII was not expected to be involved in the attachment and entry or post-entry intracellular trafficking. The L(192)F and S(193)F reduced HDV G RNA levels by ~12.8-fold and 3.3-fold, respectively, and also did not have a substantial effect on the fraction of the HDV-infected cells (Fig. 5 and 6). For TMDIV, the S(204)R, Y(206)H, Y(206)N, L(213)I, and C(221)G considerably reduced the HDV infectivity, which was reflected by a decrease in the levels of HDV G RNA by ~3-fold to 7-fold and in the fraction of the infected cells by ~3-fold to 5-fold. Only for Y(206)H and L(213)I, we did not observe a considerable reduction in the fraction of the infected cells. Interestingly, the finding that TMDs II, III, and IV can regulate HDV infectivity could suggest that different domains of HBsAg are likely work in concert during the infection, and alterations in the domains that are not exposed on the outside of the virions and are also not directly involved in the interactions with HDV RNP could have short- and long-distance effects on the HBsAg sequences, which are directly involved in the attachment, entry, trafficking, and/or uncoating, and thus considerably impact the overall levels of the infectivity of HDV virions. This opens an interesting new avenue of research, which should focus on revealing the mechanisms of how the TMDs can substantially but indirectly influence the aspects of the infectivity of HDV, that is expected to advance our understanding of the important intramolecular interactions within HBsAg molecules.

The remaining impactful DRMs were located in the MAL. As being exposed on the outside of the virions, the MAL residues can possibly participate in both attachment/entry and trafficking. It was found that the K(160)N, F(161)L, and W(172)C diminished HDV G RNA levels by ~3.2-fold to 5.4-fold, and only K(160)N also reduced the fraction of the infected cells by about 3.6-fold (Fig. 6). An earlier report, which was examining the mutations K(160)A, F(161)A, and W(172)A, found the reduced accumulation of HDV RNA genomes in infected cells for K(160)A only (31), which shows that for these particular positions in the MAL, the effect of the mutations can depend on to which amino acid the original residue was changed. The above-described examples, when DRMs only affected the levels of HDV G RNA but not the fraction of the infected cells, could possibly indicate that in those cases the main DRM-mediated functional impact occurred during post-entry event(s).

## DISCUSSION

This study was specifically focused on the effects of the DRMs in the HBsAg on the assembly and infectivity of HDV. The drug resistance mutations initially were selected in the HBV polymerase ORF, which overlaps with the ORFs for the viral envelope proteins, and therefore causes the corresponding amino acid substitutions in HBsAg (7–27). The human HDV is the natural sub-viral agent of HBV, which employs the HBV envelope proteins to form its own virions and to infect hepatocytes using the HBV receptor (1–6). Accordingly, the DRMs occurring in HBsAg were expected to have an impact on the formation/egress of HDV virions and/or their infectivity. As shown in Fig. 3, the DRM-bearing variants of HBsAg facilitated diverse levels of the HDV assembly, while none of the DRMs was able to completely abolish the generation of secreted HDV virions. Furthermore, 17/30 tested DRMs did not exert a considerable influence on the assembly of HDV virions. The other 13/30 tested DRMs notably reduced the levels of the assembly of HDV virions by at least 3-fold as compared to the levels of HDV assembly facilitated by the corresponding wt HBsAg not bearing DRMs (Fig. 3). Therefore, our study obtained evidence that the drug resistance mutations in the HBV sequence were capable of considerably reducing the HDV assembly.

Our analysis further suggested that for the five DRMs, Y(72)C, R(169)G, V(194)F, I(195)M, and W(196)R, the considerable contributing factor for decreased HDV assembly in the majority of the cases was most likely the decreased yield of secreted HBsAg (Fig. 3 and 4). It needs to be noted, however, that our results were in agreement with the previous reports, which showed that often there was no direct correlation between the yields of secreted HBV envelope proteins and the levels of egressed HDV virions, and that the productivity of the HDV assembly in a large part depends on the HBsAg properties rather than just on the yield of HBsAg ([31, 32, 35, 36], and Fig. 3 and 4). For the other six cases, the primary reason for diminished HDV assembly most likely was the altered (by a specific DRM) properties of HBsAg, and not the levels of HBsAg. In this group, the DRMs, W(196)L, W(196)V, and W(196)A, which resided in the HDV-BS, likely impaired the interactions between the large delta antigen (in the context of the HDV RNP) and the HDV-BS (in the context of the HBV envelope proteins). This resulted in an altered process of the formation of HDV virions (i.e., envelopment of HDV RNP with HBV envelope proteins) (Fig. 3 and 4). For the other three DRMs in this group, L(192)F, Y(206)H, and Y(206)N, which (i) are resided in the TMDs and are not located in the HDV-BS and (ii) thus are not directly involved in the formation of HDV virions, the decrease of HDV assembly was primarily facilitated by indirect mechanism(s) mediated likely by the altered functional properties of the DRM-bearing HBsAg that impacted either the formation of HDV virions and/or their secretion. Finally, it appears that for the two DRMs, W(196)S and Y/F(161)L, both the altered properties of the mutated HBsAg and decreased yield of DRM-bearing envelope proteins likely contributed to the diminished yield of HDV virions (Fig. 3 and 4). It is worth noting that out of the above-mentioned impactful DRMs, only the ones located in the HDV-BS (Fig. 1 and 2) directly affected HDV assembly, while the effect of the other DRMs on the yield of HDV virions was mediated by indirect mechanism(s), which are yet to be fully understood.

Some of the observed effects were HBV genotype-specific. Thus, for example, the L(192)F located in TMDIII considerably reduced HDV assembly by more than 3-fold only for the genotype D of HBV. The Y(206)H and Y(206)N decreased HDV assembly also by about 3-fold for the HBV genotype B only, and not for the genotypes C and D. There were also some HBV genotype-specific differences in the effects on the HDV assembly mediated by the DRMs residing in the LCL and in the HDV-BS (Fig. 3).

In summary, we determined that DRMs in HBsAg could considerably decrease the yield of secreted HDV virions, and sometimes such observed DRM-mediated effects were HBV genotype-specific (Fig. 3). It is also important that our study discovered that certain amino acid residues of the TMDs III and IV were capable of influencing the assembly of HDV (Fig. 2 and 3). The considerably decreased levels of circulating HDV virions *in vivo* could (i) negatively impact the continuous spread and superinfection of HDV, which are the critical determinants of the establishment and maintenance of chronic HDV infection (40–43), and (ii) also reduce the HDV reservoir in the infected liver. The latter could, in turn, reduce the burden of HDV-related liver pathogenesis. The follow-up studies are warranted to determine the mechanism(s) by which certain DRMs resided in the positions of HBsAg that are not directly involved in interactions between the HDV RNP and HBsAg were capable of indirectly greatly diminishing the HDV assembly levels.

Furthermore, it was very interesting to find out that the majority (23/30) of the tested DRMs considerably (≥3-fold) decreased the infectivity of the HDV virions coated with DRM-bearing HBsAg (Fig. 5 and 6). This group of the impactful DRMs includes all above-mentioned 13 DRMs, which affected the levels of HDV assembly (Fig. 3 and 6). Four DRMs completely abolished the infectivity of HDV virions (Fig. 5 and 6). Two of these DRMs, I(195)M and W(196)R, located in the HDV-BS, which participates in the direct interactions between the large delta antigen and HBsAg, most likely critically impaired the process of the intracellular post-entry uncoating of HDV virions, during which the HDV RNP loses the envelope, including the HBV envelope proteins. The Y(72)C residing in the LCL cannot be involved in the attachment/entry and the virion’s trafficking, because LCL is inside the virion. It is also not involved directly in the HDV RNP-HBsAg interactions, because it is not a part of the HDV-BS (Fig. 2). Therefore, the abolishing of the HDV infectivity by this DRM is most likely mediated by an indirect mechanism, which is yet to be uncovered. Given the relatively close location of this DRM to the TMDII, the inhibitory mechanism may involve long-distance influence on the functioning of the MAL during the attachment/entry or trafficking, while other possibilities also cannot be excluded at this point. Curiously, one previous report concluded that the LCL apparently does not bear the infectivity determinants for HDV, while the authors acknowledged that they were only able to test the positions, mutations of which were sufficiently permissive for the formation and secretion of HBsAg-bearing SVPs. Among the mutations that critically impaired HBsAg secretion was Y(72)A, and since for this mutation secreted L and S envelope proteins were barely detectable, the authors did not attempt to produce and to examine the properties of the HDV virions coated with Y(72)A-bearing HBsAg (30). The fourth mutation R(169)G was located on the outside of the virion in the MAL and in close vicinity to the TMDIII (Fig. 2), and it could be involved in the functioning of the MAL during the attachment/entry and/or trafficking, while the exact mode of action leading to the loss of the infectivity caused by this DRM is yet to be elucidated. Thus, the follow-up studies are warranted to uncover the detailed mechanisms by which the above-mentioned four DRMs mediated the loss of HDV infectivity.

It was reasonable to anticipate that DRMs located in the HDV-BS (Fig. 1 and 2) and therefore likely can impact direct interactions between HBsAg and large delta antigen of HDV RNP, which occur during the post-entry uncoating of the virions inside the infected cell, will have a major impact on the HDV infectivity. This happened to be the case, and all DRMs residing in the HDV-BS markedly diminished HDV infectivity, which was reflected by substantial reduction of (i) the levels of HDV G RNA in the infected cells and (ii) the fraction of the HDV-infected cells (within the ranges from 3.2-fold to 437.1-fold and from 3.1-fold to 108.8-fold, respectively) (Fig. 5 and 6). With the exception of the two already mentioned above DRMs I(195)M and W(196)R in the HDV-BS that facilitated the total loss of the infectivity, the most profound effect on the HDV infectivity was exerted by all tested DRMs located at position 196, that is, W(196)S/L/V/A (Fig. 5 and 6). At the same time, the V(194)F and M(198)I affected HDV infectivity to a lesser extent. As explained above, because HDV-BS is located inside the virion (Fig. 1 and 2), it cannot have a direct impact on the attachment/entry and on the post-entry trafficking, but it is directly involved in the uncoating. Therefore, the primary effect on the infectivity here is likely mediated by altered uncoating, leading to a considerable reduction of the number of HDV genomes that post-entry could reach the nucleus and start HDV genome replication. Since it appears that the role of HDV-BS in mediating the HDV virions’ infectivity was not specifically addressed in detail previously, and we have only limited data suggesting that natural mutants in this region could affect the infectivity ([32], and the data of this study), further studies should focus on investigating the detailed mechanism of how the amino acid residues, which form the HDV-BS, facilitate the infectivity of HDV virions.

The remaining DRMs that reduced HDV infectivity were located in the MAL and in the TMDII, TMDIII, and TMDIV. As mentioned above, only residues of the MAL are located outside the virions and therefore could affect the attachment/entry and/or intracellular trafficking. We speculate that the DRMs F(161)L and W(172)C, which only resulted in a considerable reduction of the levels of HDV G RNA, and did not have a great effect on the fraction of the infected cells (Fig. 5 and 6), could possibly alter the trafficking and not the attachment and entry. While since the third impactful DRM in the MAL K(160)N resulted in substantial reduction of both the levels of HDV G RNA and the fraction of the infected cells (Fig. 5 and 6), it may possibly affect the attachment/entry, and the trafficking as well. Such interpretations, however, should be further tested in the follow-up studies.

The transmembrane domains II, III, and IV are not exposed on the outside surfaces of the assembled virions and are not parts of the HDV-BS, and thus are not expected to have a direct involvement in influencing the attachment/entry, post-entry trafficking, or virion’s uncoating. This, at least in part, explains why these domains were not previously investigated in terms of their possible role in regulating the infectivity of the virions, while other regions of HBsAg have been examined quite extensively (1, 4, 30–32, 35, 36, 39, 44–46). Our analysis revealed very surprisingly that certain residues in each of the above-mentioned TMDs substantially decreased the overall infectivity of HDV virions by considerably (by ≥3-fold) reducing either only the accumulation of HDV G RNA in the infected cells or both, the levels of HDV G RNA as well as the fraction of the HDV-infected cells (Fig. 6). The reasons for these observations are not immediately apparent, but the results suggest a very interesting hypothesis that different domains of the HBsAg work in an intramolecular coordinating manner regulating the assembly and infectivity of the virions, and that there exist certain short- and long-distance interactions between different regions of the HBsAg, some of which are being affected by DRMs. It also became apparent that the functioning of some distant domains may have a critical but indirect impact on certain functions, which are mediated by other HBsAg regions that are directly involved in the attachment/entry, trafficking, and/or uncoating. In agreement with this theory, we found that several DRMs in the TMDs III and IV, which were located either right next to the HDV-BS or very close to it (i.e., L(192)F, S(193)F, S(204)R, Y(206)H, and Y(206)N), had a substantial effect on the HDV infectivity. These observations suggested that the above-mentioned DRMs could cause the structural alterations of the TMDs, which could, in turn, affect the functioning of the HDV-BS in terms of facilitating the interactions between HBsAg and large delta antigen of the HDV RNP. Clearly, the new data reflecting the involvement of TMDs II-IV in regulating the virions’ infectivity strongly suggest that additional further studies are necessary to uncover the mechanisms of how these domains can influence the functions of the virions related to their overall infectivity. In addition, further research may also find that the same DRMs could cause somewhat different functional consequences in the context of different HBV envelope proteins, the L, M, and S (Fig. 1). Interestingly, different functional impacts of the same mutation in the context of different HBV envelope proteins were noted previously (30).

In the settings of natural HDV infection, the DRM-mediated considerably decreased levels of the HDV assembly and greatly reduced or abolished HDV infectivity (Fig. 3, 5 and 6) could potentially impair the spread and superinfection of HDV, which serve as critical determinants of the establishment and maintenance of chronic HDV infection (40–43). The important anticipated consequence of the impaired spread/superinfection will be the shrinking of the HDV reservoir in chronically infected liver that could reduce HDV-related pathogenesis. Importantly, the four above-mentioned DRMs that resulted in the loss of the infectivity of HDV virions, Y(72)C, R(169)G, I(195)M, and W(196)R, could, in theory, facilitate the sustained suppression or even possible clearance of chronic HDV infection. Such effects of the DRMs are likely feasible if these DRMs are present on all or on the predominant majority of the HBsAg molecules, which are produced by the HBV genome replication or derived from the 5′-HBV-human-3′ RNA species that are transcribed from integrated HBV DNA independently of the HBV replication. The latter source of HBsAg *in vivo* could supply the predominant majority of the HBV envelope proteins during chronic infection (41–43, 47). In terms of the source of the DRM-containing envelope proteins, it needs to be considered that (i) there are HBV genomes in patients that contain pre-existing DRMs (48–50), which could remain in the pool of the replication-derived mRNAs for HBsAg, and also could reside among the HBV DNA integrants generating 5′-HBV-human-3′ RNAs encoding HBsAg, for which the double-stranded linear (DSL) HBV genome was produced from the DRM-bearing variants, although usually pre-existing DRM-bearing genomes do not represent a major fraction of HBV genomes in the subject; (ii) during the antiviral treatment of individuals chronically infected with HBV, there will be a selection for the genomes bearing DRMs, and the fraction of such DRM-bearing genomes in the pool of the replicating HBV genomes could be substantial or predominant (up to 98%–100% of all HBV genome sequences recovered from each tested patient) (48, 50), in which case the predominant majority of HBsAg molecules will likely contain DRMs; (iii) the HBV DNA integrations accumulate over the entire course of chronic HBV infection (51–53), and during the replication of the above-mentioned predominant genomes with DRMs (48, 50), the integration will continue and the majority of the generated DSL genomes at that time will bear DRMs and the majority of the newly integrated HBV DNA species will bear DRMs as well and they can produce DRM-bearing HBsAg (54) independently of the HBV genome replication at any later stage of the infection; and (iv) for each particular HBV-infected individual and for a certain stage of the chronic HBV infection, the fraction of DRM-bearing HBsAg will depend on the ratio of HBsAg mRNAs with and without DRMs (regardless of the origin of such mRNAs). Thus, further studies examining the relative fractions of the DRM-containing HBV DNA integrants and also of DRM-bearing mRNAs for HBsAg per patient using large cohorts of individuals chronically infected with HBV are warranted to better understand the potential extent of the impact of DRMs on the functional properties of the entire pool of HBsAg molecules in each subject.

Another aspect that should be considered is that in chronically infected patients, the DRMs can be present as individual single mutations or exist as a combination of mutations (13, 55–60). The percentage of patients bearing a single DRM in the infecting HBV can represent a substantial proportion of the total population of subjects with DRMs (both—with a single DRM and with multiple DRMs). Thus, it has been previously demonstrated that the fraction of individuals with single DRMs could be quite considerable: (i) the study conducted in Botswana reported 24.6% of HBV-infected subjects as single DRM bearers among all tested patients containing DRMs (58), (ii) another study examining Turkish patients with chronic hepatitis B reported 36% of single DRM bearers (59), and (iii) a different study that analyzed a very large cohort of HBV patients in Southwest China reported 57.64% of the single DRM bearers (60). Therefore, these are two natural situations, and thus examining the effects of the single DRMs on the viral properties (HDV in this case) represents biologically relevant and important research. In addition, the detailed analysis of the effects mediated by the combinations of DRMs is not expected to be done without analyzing the effects that are mediated by single DRMs that are present in the particular natural DRM combinations. It needs to be thus noted that our findings in terms of the influence of natural individual DRMs on the assembly and infectivity of HDV built the foundation for further research, which will focus on the natural DRMs combinations and on the analysis of the functional impact of the entire combination of the DRMs versus the impact of each individual DRM.

Overall, this report demonstrated that DRMs located in HBsAg could substantially affect the HDV life cycle by considerably decreasing the yield of secreted HDV virions and by markedly reducing the infectivity of HDV virions (by ≥3-fold), of which the latter was the major impact of the DRMs. Furthermore, our study showed that the transmembrane domains II, III, and IV can critically regulate the infectivity of assembled HDV virions. Therefore, our new findings certainly advance our understanding of (i) how the HBV-specific drug treatments affect the HDV life cycle, (ii) the mechanism of HBV-HDV interactions, and (iii) the roles of the HBV envelope proteins in facilitating the assembly and infectivity of the HDV virions.

## MATERIALS AND METHODS

### Vectors containing the DRMs in the HBsAg

The DRMs in HBsAg investigated in this study are presented in Table 1. There were a total of 30 DRMs tested. They were located in the LCL, TMDII, MAL, TMDIII, HDV-BS, and TMDIV (Fig. 1 and 2). To introduce the DRMs in HBsAg of the three HBV genotypes, B, C, and D, we used our previously described constructs expressing the envelope proteins of HBV: (i) pNF-HBV-LMS-B2 (for genotype B of HBV; the accession number for this HBV variant was AB205119), (ii) pNF-HBV-LMS-C5 (for genotype C; the accession number for this HBV variant was AF411411), and (iii) pNF-HBV-LMS-D1 (for genotype D; the accession number for this HBV variant was AB205127) (32). The plasmids pNF-HBV-LMS-B2 and pNF-HBV-LMS-D1 each express the L, M, and S envelope proteins. The vector pNF-HBV-LMS-C5 expresses only the L and S envelope proteins because it is based on the sequence of the natural HBV variant that is the M-minus mutant. The constructs pNF-HBV-LMS-B2 and pNF-HBV-LMS-D1 did not contain any DRMs, and, therefore, they were used as the vectors expressing the wt HBsAg of the corresponding HBV genotypes. The construct pNF-HBV-LMS-C5 has been modified to contain S instead of R in position 204 (numbering is for the S envelope protein), which eliminated the DRM S(204)R that was already present in the natural variant of the HBV genotype C, which was used to generate this vector. The modified construct was named pNF-HBV-LMS-C5wt, and it was then used as the wt HBsAg-producing plasmid for genotype C. Next, the DRMs were introduced in the vectors pNF-HBV-LMS-B2, pNF-HBV-LMS-C5wt, and pNF-HBV-LMS-D1 using the site-directed mutagenesis kit QuickChange II (Agilent). For each of the three HBV genotypes, we made 30 different HBsAg-expressing vectors, each bearing a single DRM.

### Transfections

To produce HDV virions coated with HBsAg of different HBV genotypes (B, C, or D) and either not bearing (wt) or bearing the DRMs, Huh7 cells were co-transfected (in a 1:1 ratio) with the plasmid pSVLD3 initiating HDV RNA genome replication and a construct expressing HBsAg of a particular HBV genotype with or without a DRM in it using Lipofectamine 2000 (Invitrogen) (32, 33). The plasmid pSVLD3 contains the sequence of genotype 1 of HDV (M21012). For measuring the levels of secreted HDV virions, the media from the transfected cells were collected at day 9 post-transfection and assayed for the copy numbers of HDV genomic RNA via RT-qPCR (32–34). The same harvested media were also used for the analysis of secreted HBsAg.

### Infection of HepG2-NTCP cells using HDV virions

The HDV virions for infection were generated using the co-transfection strategy in Huh7 cells as described above (32, 33). For the analysis of the HDV virions’ infectivity, the DRMs in the context of genotype D of HBV were examined. The media from co-transfected Huh7 cells was harvested during the time period from day 7 to day 10 post-transfection, and then concentrated using polyethylene glycol (PEG) (Fisher) as it was described previously (33, 38). The HDV infectivity was investigated using *in vitro* infection of HepG2-NTCP cells expressing on their surface the HBV receptor NTCP (3, 37). The HepG2-NTCP cells were a gift from Dr. Guangxiang Luo. The HepG2-NTCP cells were cultured in 48-well plates, the bottom of which was coated with rat tail collagen (Sigma), using advanced DMEM/F12 cell culture medium (Gibco) supplemented with 10% fetal bovine serum (FBS), 2 μg/mL puromycin, 4 mM L-glutamine, and 1× antibiotic/antimycotic solution. The infection medium, which consisted of DMEM medium (Gibco) supplemented with 1% DMSO, 5 μg/mL hydrocortisone solution, and 1× antibiotic/antimycotic solution, and which also contained HDV and 5% PEG, was incubated with HepG2-NTCP cells overnight. After the incubation, the infection medium was removed from the cells and replaced with DMEM medium (Gibco) supplemented with 10% FBS, 2 mM L-glutamine, 1% DMSO, 5 μg/mL hydrocortisone solution, and 1× antibiotic/antimycotic solution, which did not contain PEG or HDV. We measured two parameters of the infectivity of HDV virions: the levels of HDV genomic RNA (HDV G RNA) in the infected cells and the fraction of the HDV-infected cells. For measuring the levels of HDV G RNA, the cells were infected using a multiplicity of infection (MOI) of 70 (i.e., MOI was 70 HDV GE/cell in the inoculum). For examining the HDV-infected cells by IF and determining the fraction of the infected cells, the infection was done using the MOI of 100. The infected HepG2-NTCP cells were harvested on day 8 post-infection, and then were used (i) for the isolation of total RNA using TRI reagent, or (ii) for the immunofluorescence.

### Analysis of secreted HBsAg

The levels of secreted HBsAg in the media collected from transfected Huh7 cells were measured using HBsAg-specific ELISA (Abbexa).

### RNA isolation for RT-qPCR

Total RNA was extracted from infected HepG2-NTCP cells or from the media of transfected Huh7 cells using TRI Reagent (Molecular Research Center). The isolated total RNA was then treated with 7 U of TURBO DNase (Fisher) for 1 h at 37°C. DNase-treated RNA (dtRNA) samples were re-extracted with TRI reagent and used for cDNA synthesis employing LunaScript RT Master Mix Kit (Primer-free) (New England Biolabs) (61, 62).

### Analysis of intracellular and secreted HDV genomic RNA using RT-qPCR

The HDV-specific qPCR assay amplifying the sequences of HDV genomic (G) RNA (HDV G RNA) used the forward primer (312-GGACCCCTTCAGCGAACA-329), the reverse primer (393-CCTAGCATCTCCTCCTATCGCTAT-360), the TaqMan probe (5′-FAM (6-carboxyfluorescein)-332-AGGCGCTTCGAGCGGTAGGAGTAAGA-357-3′-BHQ-1 (Black Hole Quencher−1) (HDV positions’ numbering was according to Kuo et al. [63]), and TaqMan Gene Expression Master Mix (Fisher). The reverse primer was also used for the synthesis of cDNA. The copy numbers of HDV G RNA were calculated by using 10-fold dilutions of *in vitro*-transcribed and gel-purified unit-length HDV G RNA standard (the range was from 200 to 20,000,000 GE of HDV) (34, 38).

### Analysis of infected (HDV-positive) HepG2-NTCP cells using IF

The HepG2-NTCP cells were fixed with 4% paraformaldehyde, washed two times with PBS, and then permeabilized using 0.2% Triton X-100. The polyclonal mono-specific rabbit antibodies (GenScript) raised against the peptide, which sequence EGGSRGAPGGGFVPNLQGVPESPFSRTGE was present on both small and large forms of the delta antigen (HDV genotype 1, M21012) and have been used previously by others for producing delta antigen-specific antibodies (64), were employed for staining for the delta antigens (small and large) for the identification of the HDV-infected HepG2-NTCP cells. To stain the cells for human alpha-tubulin, we used mouse monoclonal antibodies (Sigma). To stain for nuclear DNA, we used DAPI (4′,6′-diamidino-2-phenylindole). Stained for the above-described three markers, HepG2-NTCP cells were analyzed using an inverted Nikon TE2000-U microscope with a 20× objective. By using the staining for human alpha-tubulin and for nuclear DNA, the total number of cells was measured. By using the staining for the delta antigens, the number of HDV-infected HepG2-NTCP cells was quantified. The percentage of the HDV-infected (i.e., positive for the delta antigens) HepG2-NTCP cells was then calculated by dividing the number of the HDV-positive cells by the total number of cells.
